# The cycle of solitude and avoidance: a daily life evaluation of the relationship between internet addiction and symptoms of social anxiety

**DOI:** 10.3389/fpsyg.2024.1337834

**Published:** 2024-01-22

**Authors:** Cristóbal Hernández, Martín Ferrada, Joseph Ciarrochi, Sergio Quevedo, José Antonio Garcés, Raimundo Hansen, Baljinder Sahdra

**Affiliations:** ^1^Escuela de Psicología, Universidad Adolfo Ibáñez, Santiago, Chile; ^2^Instituto Milenio para la Investigación en Depresión y Personalidad, MIDAP, Santiago, Chile; ^3^Institute for Positive Psychology and Education, Australian Catholic University, North Sydney, NSW, Australia

**Keywords:** social anxiety, internet addiction, loneliness, avoidance, ecological momentary assessment

## Abstract

A consistent association has been observed between internet addiction and symptoms of social anxiety. However, there is a lack of empirical research that delves into potential explanations for this relationship and its directionality, making it difficult to translate findings into development of interventions for social anxiety that account for technology-related behaviors. The present study aimed to evaluate the longitudinal dynamics between internet addiction, symptoms of social anxiety, avoidance of social interactions, and using the internet to cope with loneliness. By means of an ecological momentary assessment study, we evaluated a sample of 122 young adults from Chile using intensive self-report measurements five times a day, for a period of 10 days. Using mixed-effects models, we examined the directionality between internet addiction and symptoms of social anxiety, together with an explanation of their relationship. Results indicate that internet addiction antecedes symptoms of social anxiety; however, the reverse relationship was not observed. Furthermore, instances where individuals avoided social interactions or used the internet to cope with loneliness were predictive of later increases in levels of internet addiction, suggesting a vicious cycle. Significant heterogeneity was observed in these effects, highlighting the need for a more personalized approach when including technology-related behaviors in social anxiety interventions. Theoretical and clinical implications are discussed.

## 1 Introduction

Internet connections have become an integral part of modern society because of the numerous benefits they provide to individuals, organizations, and entire economies. However, these evidently positive effects of the internet are also sometimes associated with negative consequences, such an uncontrolled internet usage. Internet addiction is an umbrella term (Starcevic and Aboujaoude, 2017) defined as difficulty regulating one’s internet usage associated with negative consequences, often involving compulsive and withdrawal symptoms, rumination about being online when offline, and disengagement from daily activities (Young, 1998; Ciarrochi et al., 2016; Hsieh et al., 2021).

People suffering from such difficulties to control the time they spent online have an increased likelihood for social anxiety disorder (Turgeman et al., 2020; Cai et al., 2023; Wei et al., 2023), which has an estimated prevalence of 4% worldwide (Stein et al., 2017) and is characterized by intense fear or avoidance of social interactions and situations that involve the possibility of being scrutinized (American Psychiatric Association, 2013). While the association between Internet addiction and social anxiety has been identified, there remains a significant gap in the literature regarding the directionality of this relationship, hindering the translation of research into practice.

Several studies have found a link between internet addiction and social anxiety in adolescents and young adults (Tras and Gökçen, 2020; Turgeman et al., 2020; Zhang et al., 2020; Küçükal and Sahranç, 2022). However, most research findings about internet addiction and its correlates tend to rely on cross-sectional samples (Saadati et al., 2021). As one important aspect of internet addiction is that it often involves a complete immersion in the task at hand (Mazzoni et al., 2016), it is likely that internet addiction may make social anxiety worse (Lee and Stapinski, 2012; Fumero et al., 2018) as while on the internet, a person might not participate in other activities that could be more helpful to reduce symptoms of social anxiety such as practicing social skills or socializing.

A common and generally accepted conceptualization of social anxiety is that it is partly maintained through avoidance of feared situations (Clark and Wells, 1995; Wong et al., 2014) in which an individual may feel exposed to potentially painful social rejection or criticism (American Psychiatric Association, 2013). Consequently, treatment usually involves some degree of exposure to feared situations (Parker et al., 2018; Horigome et al., 2020). As social interactions require practice, it is likely that a lack of them may foster fears about one’s social performance. In fact, a recent systematic review showed a modest increase in social anxiety during the COVID-19 pandemic (Kindred and Bates, 2023), a period characterized by decreased face-to-face social interactions. As such, spending too much time on the internet may discourage engaging in challenging and rewarding social interactions, potentially making social anxiety worse by hindering exposure to social situations.

If internet addiction has negative consequences for physical (Zheng et al., 2016; Alimoradi et al., 2019; Wang et al., 2022; Zhou et al., 2022) and mental health (Ko et al., 2012; Veisani et al., 2020; Huang, 2022; Wang et al., 2022), an important question is why some individuals lose control over their internet usage.

The compensatory internet use model proposes that addiction arises because the internet is used to fulfill real-world needs in a virtual environment (Kardefelt-Winther, 2014). If this is not accompanied by other efforts to address such needs, relying solely on the internet may worsen the situation, creating the conditions for addictive behavior to emerge. As such, behaviors such as internet gaming, social media usage, or browsing information of interest may be used to “escape” or decrease negative emotional experiences (Griffiths, 2005). For example, using the internet to distract oneself from worry has been shown to predict increased levels of internet addiction in daily diary studies (Hernández et al., 2022). In the case of social anxiety, the internet may serve as a tool to compensate for the lack of social interactions, addressing loneliness or avoiding social interactions altogether (Erwin et al., 2004; Weidman et al., 2012 Wang J. L. et al., 2019 O’Day and Heimberg, 2021). If this is the case, it is likely that social anxiety itself does not directly explain internet addiction. Instead, the specific behaviors associated with using the internet to avoid social interactions or cope with feelings of loneliness may be stronger predictors of internet addiction.

Regarding social avoidance, as the internet provides an alternative environment for social interactions with a higher degree of perceived safety and control, it allows more socially anxious individuals to engage socially while keeping their triggers of anxiety at bay. In the most favorable cases, the internet may offer them a sense of connectivity and decreased loneliness (Brand et al., 2016). However, this avoidant behavior can lead to an increased reliance on the internet for social needs and using it as an avoidant strategy that may increase the risk of it evolving into internet addiction (Caplan, 2007).

Regarding loneliness, individuals with social anxiety may isolate themselves due to the fear of social situations (La Greca and Lopez, 1998; Gross et al., 2002), leading to increased feelings of loneliness (Shaw and Gant, 2002; Caplan et al., 2009). In this case, the internet can promote social connections without direct face-to-face contact, becoming an attractive avenue for mitigating the experience of loneliness. However, reliance on online interactions as a primary social outlet can inadvertently lead to excessive use and potentially foster internet addiction (Nowland et al., 2018).

One advantage of longitudinal data for psychological processes is that it enables the separation of two sources of variation: between-person and within-person effects (Bolger and Laurenceau, 2013). In psychology, within-person variation is typically estimated in mixed-effects models, where each observation for an individual is subtracted from that particular individual’s average, commonly referred to as person-mean centering (Curran and Bauer, 2011). This alters the interpretation of the value as a difference with respect to oneself, thereby representing intraindividual change. When this separation of variability—or its equivalent—is not made before modeling the data, the resulting estimate confounds within-person variation with between-person variation (differences between individuals), consequently not clearly representing an intrapersonal process (Bolger and Laurenceau, 2013).

A specific consequence of this analytical decision is that it might not accurately represent the proposed mechanism at stake: In fact, a recent study of college students found that when the variance decomposition of the between-within-person effects was not considered, a reciprocal relationship between internet addiction and mental health symptoms emerged over time. However, when within-person variability was separated and a random intercepts model was employed, a clear pattern of unidirectional effects emerged from internet addiction to symptoms of psychological problems (Zhou et al., 2020), highlighting the practical consequences of this analytical step. Other studies that found that internet addiction predicted later mental health problems and not vice versa also used this analytical step (Ciarrochi et al., 2016; Donald et al., 2019, 2022a, b; Zhou et al., 2020).

The present study aims to evaluate the directionality of the relationship between internet addiction and social anxiety symptoms, with a focus on proximal motivations for internet use, such as avoidance and coping with loneliness online. We conducted an ecological momentary assessment to model the within-person processes that reflect daily life relationships, addressing the scarcity of research that addresses the day-to-day dynamics in which symptomatic mechanisms unfold in the context of intensive internet usage.

We had the following hypotheses:

*H1*: Symptoms of internet addiction will predict later increases in symptoms of social anxiety in daily life.

*H2*: Symptoms of social anxiety will predict later increases in symptoms of internet addiction in daily life.

*H3*: Avoiding social interactions will predict later increases in symptoms of internet addiction in daily life.

*H4*: Using the internet to cope with loneliness will predict later increases in symptoms of internet addiction in daily life.

*H5*: The effect of using the internet to cope with loneliness and avoiding social interactions on internet addiction will be stronger than the effect of symptoms of social anxiety.

## 2 Materials and methods

In the present study, we examined the daily longitudinal relationships between symptoms of social anxiety and internet addiction using an ecological momentary assessment method (EMA; Bolger and Laurenceau, 2013) in a sample of Chilean young adults.

### 2.1 Participants and procedures

We invited young adults aged 18–29 years old to participate in a study about social anxiety and internet usage through advertising on social media. We focused our study on young adults, as they have been reported to be more intensive internet users and, as such, are more likely to develop an addiction (Lozano-Blasco et al., 2022). The inclusion criteria for participation were being 18–29 years old, living in Chile, and having a smartphone with a stable internet connection. The study protocol was approved by the Committee of Ethics in science of Universidad Adolfo Ibáñez, before data collection. We provided interested individuals with a link containing an informed consent form, a study explanation, and a short demographic survey, which was answered by 1,098 individuals. We invited a random subsample balanced by sex (50% male and 50% female) to participate in the final study. We conducted a statistical power analysis using a Monte Carlo simulation with the simr package (Green and MacLeod, 2016), based on 100 simulations and data from a similar ecological momentary assessment study that associated internet addiction with depressive symptoms (Hernández et al., 2022). The simulations indicated a statistical power of 0.91 with 120 participants who responded to an average of 6 prompts each. Consequently, we aimed to recruit at least 120 participants.

We ultimately recruited a sample of 122 participants, of which 53% were women, with an average age of 23.3 years old (*M* = 23.3, *SD* = 3.06), While most of the study participants lived with their parents (58%). Demographic characteristics of the study participants can be seen in Table 1. Participants responded to an average of 29 prompts (*M* = 29.48, *SD* = 15.31), and 80% responded to at least 10 prompts, thereby ensuring sufficient statistical power for our study hypotheses.

**Table 1:** Demographic characteristics of study participants.

Variable	Category	n	%
Sex			
	Male	64	0.53
	Female	56	0.47
Lives with			
	Family/Friends	29	0.24
	Parents	69	0.58
	Partner	11	0.09
	Partner and children	3	0.03
	Alone	8	0.07
Age			
	18–21	37	0.31
	22–25	56	0.47
	26–29	27	0.23

Upon registration in the survey platform (EthicaData Services Inc., 2023), research assistants contacted each participant, explained the study in more detail and instructed them to answer short surveys five times a day, for 10 days. Participants who responded 80% or more of the prompts had an incentive of a giftcard with 10.000 CLP, and surveys were programmed at 10:00 am, 13:00 pm, 16:00 pm, 19:00 pm, and 21:00 pm.

### 2.2 Measures

#### 2.2.1 Social anxiety symptoms

Following DSM-5 (American Psychiatric Association, 2013) definition of social anxiety disorder we developed items that reflect its core features namely: (a) Marked fear or anxiety about exposure to social or performance situations, and (b) fear of negative evaluation. The four items were “Since the last beep”: “I was worried about looking clumsy when expressing myself,” “I was nervous when socializing with others,” “I felt I could have said something embarrassing when talking,” and “I avoided saying something not to embarrass myself.” All items were rated on a Likert scale ranging from 1 = *not at all* to 7 = *to a great extent*. The scores were then summed to create an indicator of social anxiety symptoms. As traditional measures of internal consistency do not apply to EMA measurements, we followed Nezlek’s (2017) recommendations and calculated a mixed effects model with three levels, where item responses were nested at timepoints, which in turn nested within individuals. Item level reliability for this construct was high (*α* = 0.86).

#### 2.2.2 Internet addiction

We selected items with the highest factor loadings from the “Emotion Dysregulation” and “Time control” subscales of the short version of Young’s (1998) Internet Addiction Test validated in Chile (Hernández and Rivera, 2018), and adapted them to an EMA format with a Likert scale ranging from 1 = *not at all*, to 7 = *to a great extent*. The three items were “Since the last beep”: “I found myself saying ‘just a couple of minutes more’ while being online,” “I tried to decrease the time I spent online however I could not make it” and “I felt bad when I was not connected to the internet.” Item-level internal consistency for the three items was low (*α* = 0.52). This low score is attributable to the fact that the Internet Addiction measure consists of two subscales, which may decrease reliability in the context of the limited number of items required for EMA settings (Nezlek, 2017). When only the first two items of the Time Control subscale were used, internal consistency increased substantially to an acceptable level (*α* = 0.75). Consequently, the Internet Addiction score was calculated based on the sum of these two ‘Time Control’ items.^1^

#### 2.2.3 Using the internet to cope with loneliness

We asked participants the extent to which they used the internet to cope with loneliness with the following question on a Likert scale ranging from 1 = *not at all* to 7 = *to a great extent*: “Since the last beep, I went online not to feel lonely.” A reliability index could not be calculated because such indices require multiple indicators.

#### 2.2.4 Avoidance of social interactions

We asked whether a participant avoided social interactions with a single question in a checkbox format: “Since the last beep, I avoided having interactions with others.” This variable was dummy coded for the analyses.

### 2.3 Data analytic strategy

#### 2.3.1 General strategy

The data were analyzed using the nlme package (Pinheiro and Bates, 2023) for the statistical environment R (R Core Team, 2023). First, we will present correlations of our study variables averaged over time, and their median and median average distances. Then, we conducted a series of longitudinal analyses using mixed effects models by means of a restricted maximum likelihood estimation (REML). We applied a within-subjects standardization for each study variable as it is reflective of within-person variation (Wang L. et al., 2019). By doing this, each measurement represents standardized deviations from a person’s average (Bolger and Laurenceau, 2013), thus reflecting intrapersonal processes.

#### 2.3.2 Formal modeling

We first created a time variable that represented each consecutive time point from the beginning of the study starting at zero (0, 1, 2,…n) so the intercept represents the dependent variable at the beginning of the study. We then created a set of models where each dependent variable was predicted by a linear function of time, and a lagged version (t-1) of our independent variables of interest to create temporal precedence in our estimates to approximate directionality. By doing this, each estimate could be interpreted on a momentary basis (e.g., moments in which someone was higher on internet addiction predicted later increases in social anxiety symptoms). We assumed a linear function of time as there was no theoretical reason to assume a more complex functional form. Intercepts and focal predictors of interest were set as random, to evaluate variability on each estimate. To justify the usage random effects, we confirmed the presence of enough heterogeneity by means of a log-likelihood ratio test. We handled missing data by listwise deletion for each model. As many psychological phenomena are not normally distributed, we applied a box-cox transformation Box and Cox (1964) to each one of our dependent variables to approximate normality of the residuals.

We calculated two models to test for the reciprocal influence between internet addiction and social anxiety symptoms: The first model predicted symptoms of social anxiety by time, and lagged values of social anxiety symptoms and internet addiction. The second model predicted internet addiction by time, and lagged values of internet addiction and symptoms of social anxiety.

We then calculated two models to test for what we defined as more proximal predictors of internet addiction: (a) A model where internet addiction was predicted by whether our participants had previously avoided social interactions in the previous time point, and (b) a model where internet addiction was predicted by the extent to which they used the internet to cope with loneliness in the previous time point.

#### 2.3.3 Effect sizes range and heterogeneity

Multilevel models can output an average estimate (i.e., the grand intercept and slopes, or *fixed effect*), and deviations of each individual from that average called *random effects* (e.g., an individual who starts the study higher in the dependent variable, and with a rate of change that is more pronounced than the average; Singer and Willet, 2003). Even though the fixed effects are usually the focus of analysis, there is valuable information in the random effects, especially in the context of longitudinal data as they allow reconstruction of an individual regression line for each participant. By doing this, it is possible to estimate a measure of the overall effect size (fixed effect) and an effect size for each participant based on their individual slopes. Consistently, and in keeping with the recent calls to characterize the heterogeneity of fixed effects, which is often under-reported (e.g., Hayes et al., 2022; Ciarrochi et al., 2023 Sahdra et al., 2023), we aimed to examine the distribution of the effect size of individual slopes of our effects of interest. The fixed effect sizes do not necessarily characterize the experience of every person in the sample. In substantive terms, this allowed us to quantify the proportion of participants who had a relatively large or small effect of, for example, internet addiction on symptoms of social anxiety. This, in turn, helped us to explore the proportion of participants for whom the effect was null, small, medium, or large, which may be useful in clinical terms to tailor interventions. Following Orth et al. (2022) we considered standardized beta values of 0.03 as small, 0.07 as medium and 0.12 were considered large.

## 3 Results

### 3.1 Descriptives and correlations

Table 2 shows correlations of our study variables averaged over time, together with their medians and median average distances (MAD).

**Table 2:** Median, MAD and Spearman’s rank correlation for the averaged study variables.

Variable	*Median*	*MAD*	1	2	3	4
1. Internet Addiction	6.67	3.28				
2. Social Anxiety	11.31	5.47	0.57**			
3. Avoidance	0.08	0.11	0.19*	0.63**		
4. Loneliness	3.32	2.01	0.65**	0.72**	0.17	

Median and MAD are used to represent median and median average distance, respectively. * Indicates *p* < 0.05. ** indicates *p* < 0.01. Internet Addiction, represents internet Addiction levels averaged over time; Social Anxiety, represents Social Anxiety levels averaged over time; Avoidance, represents Avoidance of social situations averaged over time (as a binary variable, its average is a proportion of positive cases); Loneliness, represents using the internet to cope with loneliness.

The mean scores of internet addiction were positively associated with all other variables, but they had stronger positive associations with social anxiety and coping with loneliness than avoidance. There was a strong correlation between symptoms of social anxiety, avoidance and coping with loneliness online. The correlation between avoidance and coping with loneliness was not statistically significant. Since these scores average across time, they represent between-person associations, which ignore temporal effects of within-person associations.

### 3.2 Formal models

#### 3.2.1 Internet addiction predicting later symptoms of social anxiety

*First model: From internet addiction to symptoms of social anxiety.*

**Figure 1:**
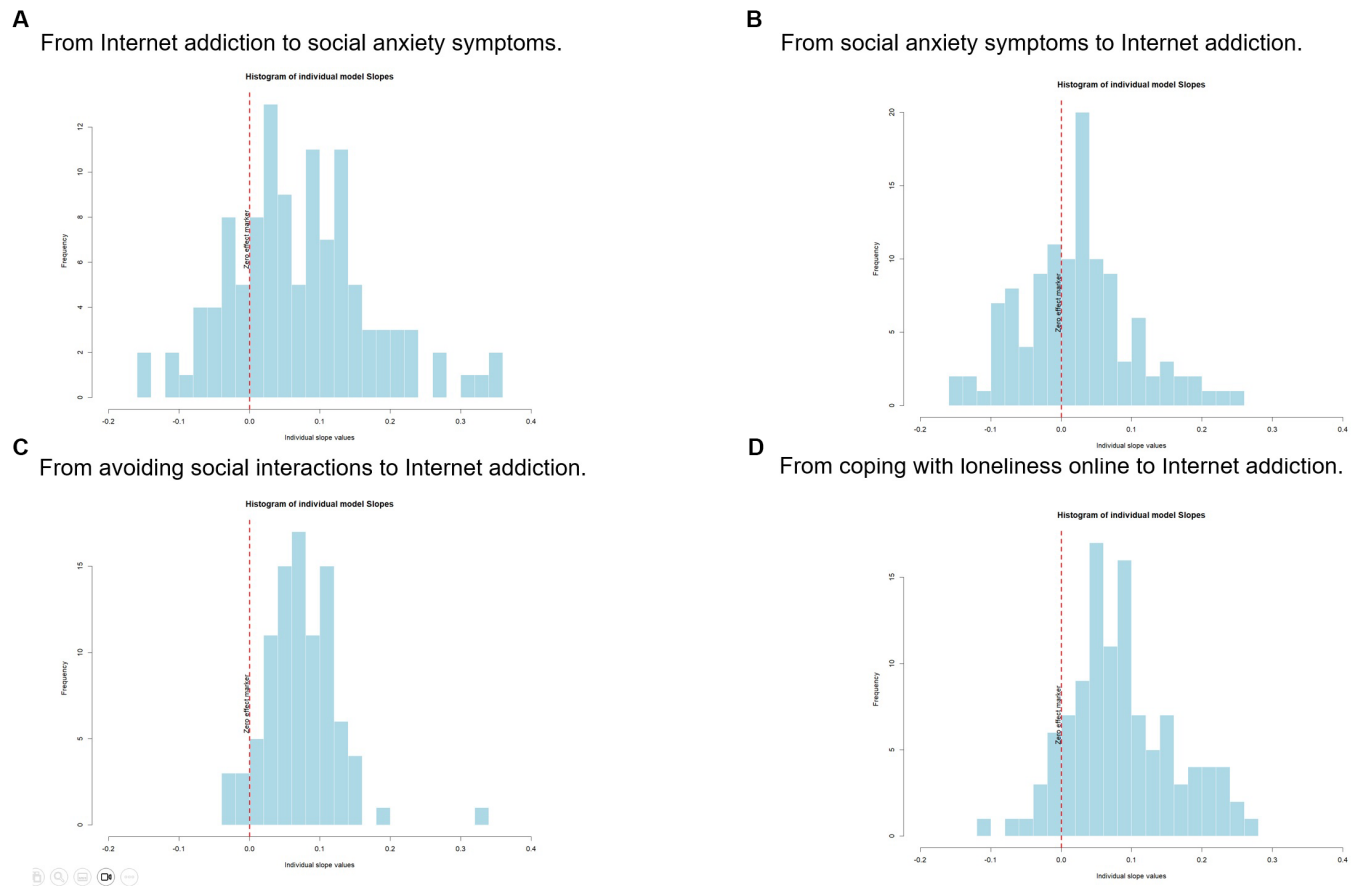
Distribution of individual effect sizes. **(A)** From Internet addiction to social anxiety symptoms. **(B)** From social anxiety symptoms to Internet addiction. **(C)** From avoiding social interactions to Internet addiction. **(D)** From coping with loneliness online to Internet addiction. Each histogram represents the reconstruction of an effect size for each participant, as standardized beta values. A vertical line indicates an effect size of zero. Values of 0.03 = small, 0.07 = medium, and 0.12 = large (Orth et al., 2022).

Lagged values of internet addiction had a positive and statistically significant effect on symptoms of social anxiety (*b* = 0.072, t (3219) = 3.107, *p* = 0.002). This effect size was considered as medium. The log likelihood ratio test was statistically significant (*p* < 0.001), indicating considerable variability. We found that 25.66% of the participants had an effect size below the threshold of being small or in the opposite direction of the expected effect, 19.46% had a small effect size in the expected direction, 23.00% had a medium effect size and 31.85% had a large effect size. The parameters for this model can be seen in Table 3, while the distribution of individual effect sizes can be seen in Figure 1A.

**Table 3:** Internet addiction predicting later values of social anxiety.

Fixed effects		Model 1: Internet addiction to Social Anxiety				
		Value	SE.	df.	*t*-value	Value of *p*
Intercept (time zero)		0.002	0.031	3,219	0.704	0.482
Linear Time		−0.002	0.001	3,219	−1.088	0.278
Social Anxiety Lag		0.198	0.017	3,219	11.581	0.000
Internet Addiction Lag		0.072	0.023	3,219	3.107	0.002
Random effects					LogLik ratio test value of *p*	
		StdDev				
Intercept		0.000				
Internet Addiction Lag		0.158			0.000	
Residual		0.942				

Only subsequent pairs of variables were entered into the model. Model 1 had 3,335 observations nested in 113 participants.

#### 3.2.2 Symptoms of social anxiety, avoidance, and coping with loneliness online predicting later levels of internet addiction

*Second model: From social anxiety symptoms to internet addiction.*

Lagged values of social anxiety symptoms did not have a statistically significant effect on internet addiction values (*b* = 0.022, t (3206) = 1.039, *p* = 0.299). The log likelihood ratio test indicated considerable variability (*p* < 0.001). In this case, 29.82% of our participants had an effect size below the threshold of being small or in the opposite direction, 37.72% had a small effect size, 18.42% had a medium effect size and 14.04% had a large effect size. The parameters for this model can be seen in Table 4, while the distribution of individual effect sizes can be seen in Figure 1B.

**Table 4:** Models predicting later values of internet addiction.

Fixed effects	Model 2: Social Anxiety					Model 3: Avoiding Social Interactions					Model 4: Coping with Loneliness				
	Value	SE.	df.	*t*-value	value of *p*	Value	SE.	df.	*t*-value	value of *p*	Value	SE.	df.	*t*-value	value of *p*
Intercept (time zero)	0.001	0.029	3,207	0.055	0.956	−0.006	0.033	2,583	−0.195	0.845	0.010	0.030	3,049	0.349	0.727
Linear Time	0.000	0.001	3,207	−0.068	0.945	0.000	0.001	2,583	0.275	0.784	−0.001	0.001	3,049	−0.378	0.706
Internet Addiction Lag	**0.142**	**0.016**	**3,207**	**8.789**	**0.000**	**0.147**	**0.021**	**2,583**	**8.319**	**0.000**	**0.123**	**0.021**	**3,049**	**7.252**	**0.000**
Social Anxiety Lag	0.021	0.021	3,207	1.039	0.299	–	–	–	–	–	–	–	–	–	–
Avoiding Social Interactions Lag	–	–	–	–	–	**0.073**	**0.021**	**2,583**	**3.466**	**0.001**	–	–	–	–	–
Coping with Loneliness Lag	–	–	–	–	–	–	–	–	–	–	**0.086**	**0.021**	**3,049**	**3.993**	**0.000**
**Random effects**				LogLik ratio test value of *p*					LogLik ratio test value of *p*					LogLik ratio test value of *p*	
	StdDev					StdDev					StdDev				
Intercept	0.000					0.000					0.000				
Social Anxiety Lag	0.134			**0.000**		–					–				
Avoiding Social Interactions Lag	–					0.103			**0.030**		–				
Coping with Loneliness Lag	–					–					0.128			**0.001**	
Residual	0.895					0.896					0.892				

Only subsequent pairs of variables were entered into the model. Model 1 had 3,324 observation nested in 114 individuals, Model 2 had 2,678 observations nested in 92 individuals, while Model 3 had 3,161 observations nested in 109 individuals. Statistically significant estimates are highlighted in bold.

*Third model: From avoidance of social interactions to internet addiction.*

Previous moments in which the participants declared avoiding social interactions had a positive and statistically significant effect on later internet addiction values (*b* = 0.736, t (2583) = 3.466, *p* < 0.001). This effect size was considered as medium, and the Log likelihood ratio test showed statistically significant heterogeneity (*p* = 0.019). Of the participants, 15.22% had an effect size below the threshold of being small or in the opposite direction, 35.87% had a small effect size, 35.87% had a medium effect size, and 13.04% had a large effect size. As a group of individuals did not avoid social interactions at all during the study period, it was not possible to compute random effects for them because they had zero variance. As such, this model was composed by a smaller number of 91 participants. The parameters for this model can be seen in Table 4, while the distribution of individual effect sizes can be seen in Figure 1C.

*Fourth model: From using the internet to cope with loneliness to internet addiction.*

Moments in which the participants used the internet more to cope with loneliness had a positive and statistically significant effect on later internet addiction values (*b* = 0.085, t (3049) = 3.993, *p* < 0.001). This effect size was also considered as medium and had a statistically significant heterogeneity according to the Log likelihood ratio test (*p* = 0.001). Regarding individual effect sizes, 19.27% of our study participants had an effect size below the threshold of being small, or in the opposite direction, 24.77% had a small effect size, and 28.44% had a medium effect size. Finally, 27.52% had a large effect size. The parameters for this model can be seen in Table 4, while the distribution of individual effect sizes can be seen in Figure 1D.

### 3.3 The relative importance of each proximal predictor of internet addiction

As symptoms of social anxiety did not have a statistically significant effect on internet addiction, but more proximal predictors did, we set to explore the heterogeneity of their effects. Our objective was to provide a clearer picture of the relationship between uncontrolled internet use and social anxiety. We thus re-coded each individual effect size as medium-and-large (> = 0.07) or small-to-null (<= 0.07) for both predictor variables. We then created a two-by-two contingency table (Table 5) with every participant with enough information to compute random effects in every variable (i.e., had enough variability for them to be computed).

Table 5 shows that for 24.42% of our study participants, avoidance and dealing with loneliness were both important in predicting later levels of internet addiction. However, for 24.41% of our sample, internet addiction was not substantially explained by the tendency to avoid or their use of the internet to cope with loneliness. For 22.09% of our study participants avoiding social interactions was the only important factor predicting internet addiction, while for 29.07%, loneliness was the only important factor explaining subsequent internet addiction levels (Table 5).

**Table 5:** Proportion of participants who had a medium and large effect size, or a small to null effect size of loneliness, avoidance, or both on internet addiction.

	Loneliness medium and large effect	Loneliness small to null effect
Avoidance medium and large effect	21 (24.42%)	19 (22.09%)
Avoidance small to null effect	25 (29.07%)	21 (24.42%)

All effects are predicting internet addiction levels.

## 4 Discussion

We investigated the relationship between internet addiction and symptoms of social anxiety in daily life. With an intensive longitudinal design and a sample of 122 young adults from Chile, we also explored individual effects and compared their relative importance.

Overall, and supporting Hypothesis 1, our results suggested that momentary increases in internet addiction levels tend to predict later momentary increases in symptoms of social anxiety. However, the opposite direction (Hypothesis 2) did not hold. On average, when individuals lose control of their internet usage, they tend to experience more symptoms of social anxiety later.

It is likely that when individuals have difficulties controlling their internet usage, they may get into a set of problems derived by not dealing with concrete life situations (Kardefelt-Winther, 2014). As social anxiety symptoms are usually maintained by avoidance (San Martín et al., 2020) which in turn may increase feelings of social inadequacy (Clark and Wells, 1995 Clark and Beck, 2012), not being able to stop spending time online may worsen social anxiety symptoms. However, even though research has usually shown that symptoms of social anxiety are associated with internet addiction (Turgeman et al., 2020; Wang et al., 2022), and even predicting it in longitudinal settings, our study results did not support the claim that social anxiety antecedes internet addiction. This discrepancy may emerge because most studies that have found said relationships are either cross-sectional (Saadati et al., 2021) or did not use within-subjects variance decomposition (Leo et al., 2021; Wei et al., 2023) which has been shown to modify regression estimates (Zhou et al., 2020).

In examining the more proximal predictors of internet usage, avoidance of social interactions, and coping with loneliness online emerged as precursor antecedents leading to internet addiction, both showing medium effect sizes. As such our findings lent support to Hypotheses 3, 4 and 5.

Regarding avoidance, moments when individuals avoid social interactions tend to predict subsequent difficulties in controlling their time spent on the internet. Similarly, our results suggest that when individuals use the internet to cope with feelings of loneliness, they are more likely to lose control of their internet usage later.

Both results are consistent with Brand et al. (2016) ‘Interaction of Person-Affect-Cognition Execution’ (I-PACE) model of internet use. According to this model, in the early stages of internet use, individuals seek gratification (Casale et al., 2016) which may include positive social experiences and entertainment. However, with repeated use, the gratification effect tends to decrease, while compensation for negative feelings (e.g., anxiety and loneliness) can become more significant factors influencing internet usage over time (Brand et al., 2016). Based on this, negative reinforcement may become a strong motivation for uncontrolled internet usage over time. This may be reasonable in the context of our study if we consider that the internet provides a medium for immediate and controlled social connectivity, thus providing relief for feelings of loneliness (Zhang et al., 2018) and anxiety related to social interactions. Consistently, a recent meta-analysis has shown a robust association between loneliness and internet addiction (Saadati et al., 2021).

Integrating across our analyses, our results suggest a vicious cycle: At the between-person level, more socially anxious individuals tend, on average, to avoid social interactions and use the internet more to cope with feelings of loneliness. At the within-person level, when individuals tend to use the internet more for these means, they also tend to have later increased difficulties controlling their internet usage. This, in turn is associated with worsening symptoms of social anxiety over time.

It is important to note that by examining the heterogeneity in individual effect sizes, we could show that elements of the described cycle did not apply to the same degree to each one of our study participants. For about 24% of our study participants, both coping with loneliness online and avoidance were relatively important in explaining their momentary internet addiction levels, and for around 24% none of them had an important effect. However, for close to 51% of our study participants, only one of these factors had an important contribution in explaining later levels of internet addiction. Also, internet addiction levels had a medium effect size on symptoms of social anxiety for 23% of our study participants, while for 32% the effect was large. These findings may indicate the existence of different profiles of emotional and behavioral dynamics relating internet usage to social anxiety, underscoring the need for a more personalized approach when developing treatment, focusing efforts, and optimizing resources (Hayes et al., 2019, 2022; Sahdra et al., 2023).

These results are also consistent with an idionomic approach (Hayes et al., 2022) in which the focus of analyses is at the level of the individual when studying psychological processes. This emphasis on individual, time-sensitive changes align with prior research on the temporal and personal nuances of behavioral responses to psychosocial stressors (Kuppens et al., 2010). For example, not everyone who uses the internet to cope with loneliness will have later difficulties controlling their internet usage, and likewise not everyone who has difficulties controlling their internet usage will have worsening symptoms of social anxiety over time. A focus on individualized effects may be a good starting point for more research into explaining individual trajectories, for example by classifying participants based on their individual effect sizes and then using that classification as a dependent variable to be explained.

Our results should be read with the limitations of our study in mind. First, the approach to our study participants was through the internet and participation was self-selected. As such, findings from this sample may not be representative of the population. However, the self-selection issue is hard to overcome in any study due to ethical reasons. Additionally, since our sample consisted solely of young adults aged 18–29 years, any generalizations beyond this age range should be made cautiously. A second problem is that despite the intensive longitudinal design and the consideration of temporal precedence and within-subject variation in our analyses, causality needs to be inferred with caution because causal inferences typically require experimental evidence (Bolger and Laurenceau, 2013). Finally, these results are best understood as daily life dynamics, and they do not represent events that happen over a longer time span. Consequently, our findings reflect short-term effects and should not be directly extrapolated to long-term dynamics. We think that different designs with varying time frames complement each other to capture the temporal dynamics representing the affective and behavioral manifestations of social anxiety and internet addiction.

Going beyond these limitations, our results provide a nuanced understanding of the temporal daily life dynamics of social anxiety and internet addiction in an increasingly technologically mediated world. While the symptoms of social anxiety themselves did not, their associated behaviors had a significant and substantive predictive value for losing control over internet use. It is likely that a focus on specific user intentions and behaviors related to internet usage may better clarify the relationship between our usage of these technologies and anxiety related disorders (Hernández et al., 2019, 2022). Accordingly, future research would benefit from focusing on the motivations driving individuals to go online, which include, but are not limited to, socializing, relieving stress, avoiding problematic situations, or seeking information to solve real-life problems. It is evident from our results that uncontrolled media usage significantly increases the likelihood of worsening social anxiety symptoms in a substantial proportion of individuals. These results are relevant for fostering an evidence-based approach to treatment development. They can help tailor known interventions for social anxiety to clients characterized by increased media and internet usage, also acknowledging the heterogeneity associated with their associated processes. They can also aid in customizing interventions for internet addiction by focusing on motivations for internet usage. This is especially important for young adults, a population with an increased risk of internet addiction (Lozano-Blasco et al., 2022).

## Data availability statement

The raw data supporting the conclusions of this article will be made available by the authors, without undue reservation.

## Ethics statement

The studies involving humans were approved by the Comité ético de Investigación, Universidad Adolfo Ibáñez. The studies were conducted in accordance with the local legislation and institutional requirements. Participants provided their informed consent electronically to participate in this study.

## Author contributions

CH: Conceptualization, Data curation, Formal analysis, Writing – original draft, Writing – review & editing. MF: Conceptualization, Data curation, Investigation, Writing – original draft, Writing – review & editing. JC: Writing – original draft, Writing – review & editing. SQ: Conceptualization, Data curation, Investigation, Writing – original draft, Writing – review & editing. JG: Investigation, Writing – original draft, Writing – review & editing. RH: Investigation, Writing – original draft, Writing – review & editing. BS: Data curation, Writing – original draft, Writing – review & editing.

## Footnotes

^1^We replicated every analysis using both versions of the Internet Addiction measure (one with three items and the other with two). The results were substantively equivalent, with only slight variations in point estimates, yet they supported the same conclusions.
